# Editorial: Design and Implementation of Rehabilitation Interventions for People With Complex Psychosis

**DOI:** 10.3389/fpsyt.2021.698432

**Published:** 2021-05-26

**Authors:** Helen Killaspy, Tom Craig, Frances Dark, Carol Harvey, Alice Medalia

**Affiliations:** ^1^Division of Psychiatry, University College London, London, United Kingdom; ^2^Institute of Psychiatry, Psychology, and Neuroscience, King's College London, London, United Kingdom; ^3^Metro South Addiction and Mental Health Services, Brisbane, QLD, Australia; ^4^Psychosocial Research Centre, Department of Psychiatry, University of Melbourne, Melbourne, VIC, Australia; ^5^Department of Psychiatry, New York State Psychiatric Institute, Columbia University Vagelos College of Physicians and Surgeons, and New York-Presbyterian, New York, NY, United States

**Keywords:** complex psychosis, mental health, rehabilitation, services, interventions

## Introduction

Between one fifth and one quarter of people who become unwell with a psychotic disorder will develop particularly complex problems (1). These include severe, treatment-resistant symptoms and cognitive impairments that affect motivation, organizational, and social skills. Co-existing mental, neurodevelopmental, and physical health conditions can often complicate recovery further, and up to three quarters have been found to be vulnerable to self-neglect and/or exploitation by others (2). Despite their high levels of need, this group has been missing from recent mental health policy internationally, resulting in inadequate treatment and, worryingly, increasing levels of institutionalization (3). The publication in 2020 of the first National Institute for Health and Care Excellence (NICE) Guideline on the mental health rehabilitation of adults with complex psychosis (4) is therefore a very welcome and important milestone, but there is an ongoing, urgent need for research to identify effective interventions for this group. In this Research Topic we aimed to collate relevant work that can help to address this evidence gap.

## Effectiveness Of Mental Health Rehabilitation

Recent decades have seen a radical transformation in how care is provided to people with severe and enduring mental health problems in many countries, with the closure of long term asylums and the development of community based services. As Dalton-Locke et al. show in their review, deinstitutionalization was largely a success and contemporary rehabilitation, comprising specialist inpatient services and supported accommodation, continue to deliver good outcomes for service users with the most complex problems, including reduced rehospitalization. However, poor quality care and institutional practices are still found in some settings and the expected progressive step-down to independent accommodation often takes considerably longer than anticipated. An alternative approach, “housing first,” developed in the US and Canada offers permanent tenancies to homeless people with mental health problems, with visiting support to assist them to maintain their tenancy. Further studies of the model focusing on those with complex psychosis are warranted.

A key objective of rehabilitation is to improve social participation (including employment). There are several well-known approaches including the Boston University Approach to Psychiatric Rehabilitation (BPR). Sanches et al. provide an interesting report of a randomized controlled trial of rehabilitation delivered by BPR trained therapists compared to an active control condition comprising mental health practitioners also focused on rehabilitation goals but without this training. Both approaches produced similar benefits. Although there are a number of plausible explanations for the lack of difference including employment opportunities in the wider economy, these results suggest that focused rehabilitation efforts are of benefit regardless of whether delivered in a particular model framework.

## Delivering Recovery-Based Rehabilitation

It is recommended that mental health rehabilitation services should provide a recovery orientated approach because it has been shown to be associated with better outcomes for people with complex psychosis (4). It is therefore very encouraging that researchers are focusing on how to do this. Two systematic scoping reviews highlighted the importance of social and environmental interventions in facilitating personal recovery. Leendertse et al. found that symptoms (affective symptoms and positive and negative symptoms of psychosis) were inversely correlated with personal recovery, while social factors (support, work, housing, and social functioning) were positively associated with it. Jaiswal et al. identified three key elements essential to personal recovery; relationships, meaning, and participation.

However, McPherson et al.'s systematic review evaluating recovery-based practice training programmes for mental health staff identified few relevant studies and little evidence for the effectiveness of the programmes that have been conducted to date. More research is clearly needed to identify how best to support staff in adopting recovery principles and two studies from the Netherlands provide further hope of a breakthrough. Zomer et al. describe the development of the “Active Recovery Triad” (ART) model, a collaborative, recovery orientated approach specifically designed for longer term mental health care settings to bring together mental health staff, service users, and family members to work together to support the individual to identify and work toward their personal recovery goals. van der Meer et al. also highlight the importance of service user involvement in the development of a complex psychosocial intervention to improve personal recovery, adopting an iterative “user centered design.” Initial pilot data for the intervention appear promising.

In keeping with the theme of service users having a key role in helping services become more recovery orientated, helpful insights into the experiences of peer support workers who were integrated into a community rehabilitation service in Australia were provided by Wyder et al. through review of their diary entries. They revealed how the peer workers used their own experiences to connect and establish trust with residents and family members and used the opportunity of engaging with residents in everyday activities to discuss informally with them their hopes and future goals. Tensions within the team about the peer workers' role were acknowledged but overall their non-clinical perspective was considered a valuable addition.

## Family And Social Supports

Positive relationships with families, friends and others are crucial for the personal recovery of people with complex psychosis. There is robust evidence to support the effectiveness of family psychoeducation in enhancing family relationships (5) but implementation has proved challenging (6, 7). Multi-family models are one of the evidence-based approaches for improving family involvement and although subject to minimal empirical study, psychodynamic versions of multi-family models have been developed widely across Italy and Latin America. In their observational study using registry data, Maone et al. describe weekly psychodynamic multi-family group sessions held in six community mental health centers in Rome, Italy, over more than 4 years. Their data suggest that it is feasible to provide and facilitate well-attended multifamily groups over the long term in an inner-city area, involving about 15% of all service users receiving treatment for severe mental illness.

A related approach, described by Tjaden et al., is that of resource groups that aim to support recovery through developing meaningful partnerships between service users and their support systems, with group meetings held between the service user, their nominated significant others and mental health professionals. Using a longitudinal case study design, the authors studied transcripts and field notes of resource groups held approximately every 3 months in the context of Flexible Assertive Community Treatment (FACT), an intensive form of community case management. Findings suggested that resource groups led to participants relating to each other in new ways and that active involvement and open communication between participants may have altered previously rigid patterns of interactions.

van Bussel et al. also aimed to improve understanding of how relationships may support or hinder recovery. Their meta-analysis examined relationships between different adolescent and adult attachment styles and symptomatic, social and personal recovery in service users with a psychotic disorder. They reported that insecure anxious and avoidant attachment are both associated with less symptomatic recovery (positive and general symptoms), and worse social and personal recovery. Whilst included studies were mostly cross-sectional and of poor quality, these findings, if replicated, may have prognostic implications as well as contributing to better treatments.

## Integrating Complex Interventions

There is growing evidence for the effectiveness of therapies that focus on functional improvements and, increasingly, this is complemented by practice-based evidence that can inform successful implementation. The case report on integrative cognitive remediation for early psychosis by Vidarsdottir et al. articulates the process of successful implementation, highlighting the core components. Having a strong evidence base for the intervention is obviously a pre-requisite, but time and investment for successful implementation are needed to ensure commitment at all levels of the organization, including the provision of adequate resources, staff training and supervision.

The study by Roeg et al. investigated the feasibility of integrating the evidence-based supported employment model, Individual Placement and Support (IPS) alongside mental health supported accommodation in the Netherlands. The study sites were eight supported housing organizations and the comparison sites were 21 mental health treatment organizations. This qualitative study found support for the feasibility and effectiveness (assessed using employment outcomes) of integrating the IPS model in both supported accommodation services and mental health organizations.

## Addressing Physical Inertia

No physical health without mental health and no mental health without physical health have become catch phrases to focus attention on the need for holistic recovery goals for people with mental illness. Rees et al. described the development of an intervention, “Action Over Inertia” (AOI), designed to address restricted activity that can be a barrier to optimal recovery for people with severe mental illness. The study was set in three residential rehabilitation facilities. This naturalistic qualitative study explored the perspectives of the participants and facilitators of AOI. The study findings drew attention to the challenges in enacting desired behavioral change for people with mental illness and the need for programs to understand and address specifically their inertia.

Alternative therapies, including mind body exercises (MBEs), have long been thought to be beneficial for general mental and physical well-being. Wei et al. in their systematic review, found modest effects on positive and negative symptoms and on depressive symptoms amongst people with schizophrenia. However, methodological problems limited their conclusions, including the fact that all the studies included compared MBEs to “treatment as usual” leaving it unclear whether the observed benefits are due to the MBE or reflect non-specific aspects of a pleasant activity led by enthusiastic coaches.

## Co-Occurring Substance Misuse

The challenges of co-occurring psychosis and substance misuse were addressed in two papers. Florentin et al. reported that in Israel, people with psychosis and substance misuse were less likely to receive mental health rehabilitation services and had higher hospitalization rates than those without co-occurring substance abuse. Their data, from 18,684 adults with schizophrenia spectrum disorders, argues for expansion of recovery services to specifically meet the needs of people with complex psychosis and substance misuse. Clausen et al.  provide evidence that suggests that Assertive Community Treatment (ACT) may be a good option to achieve this. They showed that clients with and without substance misuse problems who received 2 years of ACT had similar outcomes, with both groups achieving better housing, functioning and decreased anxiety and depression.

## Mental Health Rehabilitation And Telehealth

Finally, in the context of the global pandemic, Lynch et al.  demonstrated that recovery services could be provided virtually to people with complex psychosis. In a clinic serving New York, 90% of clients accepted telehealth sessions, including group therapies, and were able to maintain their specific treatment plans in virtual format.

## Conclusion

This Research Topic has highlighted the wide range of contemporary studies aiming to improve outcomes for people with complex psychosis. Whilst this is clearly very encouraging, most studies were in the field of mental health service interventions. We note the paucity of peer-led/co-led interventions for people with complex psychosis, and the lack of biological and pharmacological research targeting this group which also need to be addressed urgently.

## Author Contributions

HK led the drafting of this editorial. All authors contributed to the editorial and approved the final draft for submission.

## Conflict of Interest

The authors declare that the research was conducted in the absence of any commercial or financial relationships that could be construed as a potential conflict of interest.
